# Comparing early signs and basic symptoms as methods for predicting psychotic relapse in clinical practice

**DOI:** 10.1016/j.schres.2017.04.050

**Published:** 2018-02

**Authors:** Emily Eisner, Richard Drake, Fiona Lobban, Sandra Bucci, Richard Emsley, Christine Barrowclough

**Affiliations:** a Division of Psychology and Mental Health, School of Health Sciences, Faculty of Biology, Medicine and Health, University of Manchester, Manchester Academic Health Science Centre, UK; b Greater Manchester Mental Health NHS Foundation Trust, Manchester Academic Health Science Centre, UK; c Spectrum Centre for Mental Health Research, University of Lancaster, Lancaster, UK; d Division of Population Health, Health Services Research & Primary Care, School of Health Sciences, Faculty of Biology, Medicine and Health, University of Manchester, Manchester Academic Health Science Centre, UK

**Keywords:** Relapse, Psychosis, Schizophrenia, Early signs, Basic symptoms

## Abstract

**Background:**

Early signs interventions show promise but could be further developed. A recent review suggested that ‘basic symptoms’ should be added to conventional early signs to improve relapse prediction. This study builds on preliminary evidence that basic symptoms predict relapse and aimed to: 1. examine which phenomena participants report prior to relapse and how they describe them; 2. determine the best way of identifying pre-relapse basic symptoms; 3. assess current practice by comparing self- and casenote-reported pre-relapse experiences.

**Methods:**

Participants with non-affective psychosis were recruited from UK mental health services. In-depth interviews (n = 23), verbal checklists of basic symptoms (n = 23) and casenote extracts (n = 208) were analysed using directed content analysis and non-parametric statistical tests.

**Results:**

Three-quarters of interviewees reported basic symptoms and all reported conventional early signs and ‘other’ pre-relapse experiences. Interviewees provided rich descriptions of basic symptoms. Verbal checklist interviews asking specifically about basic symptoms identified these experiences more readily than open questions during in-depth interviews. Only 5% of casenotes recorded basic symptoms; interviewees were 16 times more likely to report basic symptoms than their casenotes did.

**Conclusions:**

The majority of interviewees self-reported pre-relapse basic symptoms when asked specifically about these experiences but very few casenotes reported these symptoms. Basic symptoms may be potent predictors of relapse that clinicians miss. A self-report measure would aid monitoring of basic symptoms in routine clinical practice and would facilitate a prospective investigation comparing basic symptoms and conventional early signs as predictors of relapse.

## 1 Introduction

Relapse of psychosis is common (Robinson et al., 1999) and predicts distress (Maclean, 2008), impaired vocational and interpersonal functioning (Gumley and Schwannauer, 2006), long-term deterioration (Wiersma et al., 1998) and suicide (Appleby, 1992). It frequently results in hospital admission, the single biggest expense in schizophrenia's annual UK National Health Service cost of over £3.9 billion (Almond et al., 2004, Andrew et al., 2012), the USA equivalent being $22.7 billion (Wu et al., 2005). Interventions using early signs of deterioration to prompt timely preventative action can prevent relapse (Gumley et al., 2003, Herz et al., 2000, Lee et al., 2010), but could be further developed. Predictive validity of checklists such as the Early Signs Scale (ESS; Birchwood et al., 1989) could be improved by adding other hypothesised predictors such as basic symptoms (Eisner et al., 2013, Gumley et al., 2015).

‘Basic symptoms’ are subtle, sub-clinical disturbances in one's experience of oneself and the world that prospectively predict first episodes of psychosis (FEP) (Fusar-Poli et al., 2012, Schultze-Lutter et al., 2007). Typical basic symptoms include: perceptual changes such as colours' increased vividness; mild subjective cognitive problems; decreased tolerance of stressors. Overlap between lists of conventional early signs (e.g. ESS) and basic symptoms (e.g. Schizophrenia Proneness Index, Adult Version, SPI-A; Schultze-Lutter et al., 2007) is small (< 5%). There is preliminary evidence that basic symptoms predict relapses of psychosis (Bechdolf et al., 2002, Gaebel and Riesbeck, 2014).

We aimed to investigate whether basic symptoms could be used to predict relapse in routine clinical practice and to compare them to conventional early signs in anticipation of developing and prospectively testing a basic symptoms measure. Using data from in-depth interviews, verbal checklists of basic symptoms and casenote extracts, we addressed the following research questions: 1. *Which pre-relapse experiences (early signs, basic symptoms, ‘other’) do participants report and how do they describe them?*; 2. *What is the best way of identifying basic symptoms: in-depth interview or verbal checklist?*; 3. *Which pre-relapse experiences (early signs, basic symptoms, ‘other’) are reported in casenotes?*

## 2 Methods

### 2.1 Ethics

Ethical approval was obtained from the Liverpool Central research ethics committee (ref: 12/NW/0091).

### 2.2 Which pre-relapse experiences do participants report? What is the best way of identifying basic symptoms?

#### 2.2.1 Participants

Sample A: 23 patients were purposively sampled to include a range of characteristics from three NHS (National Health Service) Mental Health Trusts between May and November 2012. Inclusion criteria were: aged over 18 years; primary clinical diagnosis of non-affective psychosis (APA, 2000); admission to crisis team or inpatient unit in the past 6 months for acute psychosis; prescribed antipsychotic medication; no illicit drug use, or alcohol abuse or dependence, during the pre-relapse period; informed consent.

#### 2.2.2 Data collection

In-depth interview: open questions explored events, feelings and experiences in the three months prior to the most recent relapse (topic guide available on request). Verbal checklist of basic symptoms: assessed experiences of basic symptoms in the three months prior to the recent relapse, based on the SPI-A, (Schultze-Lutter et al., 2007). The SPI-A (56 items) includes two overlapping lists of basic symptoms that predict FEP, ‘COGDIS’ (Cognitive Disturbances, 9 items) and ‘COPER’ (Cognitive-Perceptive basic symptoms, 14 items), in addition to 38 other basic symptoms (Schultze-Lutter et al., 2007). All interviews were audio-recorded and transcribed verbatim.

### 2.3 Which pre-relapse experiences are reported in casenotes?

#### 2.3.1 Participants

Sample A: 21/23 in-depth interview and verbal checklist participants consented to their casenotes being examined. Sample B: 187 patients (approximately 10% of those eligible) were randomly selected (stratified by clinical team) from those aged over 18 with a clinical diagnosis of non-affective psychosis (WHO, 1992) and attending Community Mental Health Teams in one NHS Mental Health Trust in November 2010. Since data was obtained from a pseudo-anonymised dataset gathered for an audit, separate ethical approval and patient consent were not required (BMA, 2014).

#### 2.3.2 Data collection

Five research assistants examined participants' electronic casenotes (n = 208) and extracted demographic information and verbatim quotations from the section of the most recent CPA review entitled “early warning signs”, “relapse indicators” or “crisis plan”.

### 2.4 Analysis

#### 2.4.1 Directed content analysis

Directed content analysis (Hsieh and Shannon, 2005) was used to quantify pre-relapse experiences. Supplementary material Section B gives details of this process. All transcripts were coded according to the stage of the relapse process being described (pre-relapse, during relapse, unrelated to relapse). Pre-relapse experiences were then coded, with codes grouped into early signs, basic symptoms and ‘other’ pre-relapse experiences. Inter-rater reliability was assessed (supplementary material Section B).

#### 2.4.2 Statistical analysis

Non-parametric statistics were used due to the relatively small size of the interview sample (see supplementary material Section B). For all analyses, findings were considered significant at p = 0.05.

## 3 Results

### 3.1 Sample characteristics

Table 1 shows demographic and clinical characteristics of the two samples.

**Table 1:** Demographic and clinical characteristics of the two study samples.

	Sample A (n = 23)		Sample B (n = 187)		Comparison of the two samples	
	Frequency	Percentage	Frequency	Percentage	χ^2^	p
Age, mean (SD), *U*	38.4	(14.0)	45.0	(11.7)	2.42	0.016
Gender, n male	11	(47.8)	116	(62.0)	1.73	0.189
Diagnosis						
Schizophrenia	17	(73.9)	159	(85.0)		
Schizoaffective disorder	6	(26.1)	18	(9.6)		
Other non-affective psychosis	0	(0.0)	10	(5.3)		
Ethnic origin					0.83	0.863
Asian or Asian British	3	(13.0)	17	(9.1)		
Black or Black British	3	(13.0)	33	(17.6)		
White British	16	(69.6)	124	(66.3)		
Other ethnic group	1	(4.3)	13	(6.9)		
Living arrangement					3.37	0.353
Family or partner	9	(39.1)	59	(31.6)		
Alone	11	(47.8)	69	(36.9)		
Shared/supported accommodation	3	(13.0)	54	(28.9)		
Homeless	0	(0.0)	4	(2.1)		
Unknown	0	(0.0)	1	(0.5)		
Level of family or carer contact					9.01	0.012
None	8	(34.8)	22	(11.8)		
Low	8	(34.8)	44	(23.6)		
High	7	(30.4)	96	(51.3)		
Unknown	0	(0.0)	25	(13.4)		

Note: descriptive statistics are N (%) and inferential statistic is χ^2^ unless otherwise specified.

### 3.2 Inter-rater reliability

Casenote data extraction: mean percentage agreement with consensus extraction was 95.7% after training and 91.4% during data collection. Stage-of-relapse coding: weighted kappa was 0.74. Pre-relapse experience coding: ICCs and kappas were calculated for three types of item (early signs, basic symptoms, other) and three types of data (in-depth interviews, verbal checklist, casenotes). ICCs all exceeded 0.72 and kappa values all exceeded 0.60.

### 3.3 Which pre-relapse experiences do participants report and how do they describe them?

#### 3.3.1 Estimated sensitivity (early signs, basic symptoms, ‘other’)

Three-quarters (74%) of participants reported ≥ 1 basic symptom, with all participants reporting both conventional early signs and ‘other’ pre-relapse experiences. Sensitivity here refers to the proportion of relapses correctly identified by a putative predictor. Since all participants in the interview sample had relapsed, it equates to the proportion reporting a particular pre-relapse experience (i.e. 74% for basic symptoms, 100% for early signs, 100% for ‘other’). No demographic or clinical characteristics listed in Table 1 significantly predicted reporting ≥ 1 basic symptom.

#### 3.3.2 Number of pre-relapse experiences reported (early signs, basic symptoms, ‘other’)

**Fig. 1:**
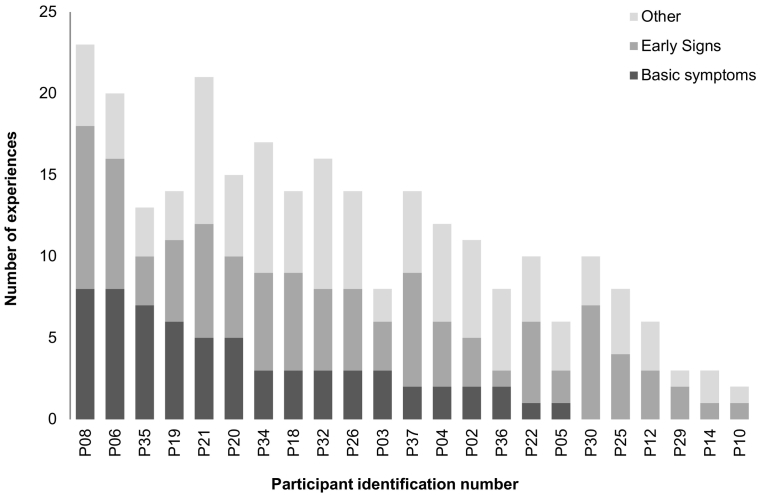
Number of basic symptoms, early signs and ‘other’ experiences reported pre-relapse in the in-depth interview or verbal checklist (n = 23), ranked by basic symptoms.

Fig. 1 shows the number of basic symptoms, early signs and ‘other’ experiences reported to begin or increase pre-relapse. Participants reported significantly more (z = 3.12, p = 0.002) early signs (Median = 5; IQR = 3,6) than they did basic symptoms (Median = 2, IQR = 0,5). However, 35% (6/17) of those reporting basic symptoms, reported at least as many basic symptoms as they did conventional early signs. Furthermore, reported pre-relapse experiences were idiosyncratic, with a wide range of experiences reported (79 experiences) and most (57%) reported by ≤ 2 participants.

#### 3.3.3 Estimated specificity (basic symptoms only)

Fourteen participants reported that they experienced basic symptoms at times unrelated to relapse (with no increase prior to relapse). Specificity, generally the proportion of non-cases correctly identified by negative test values was estimated by the proportion of the sample who did not report having experienced basic symptoms at times unrelated to relapse (39% for any basic symptoms; 70% for COGDIS; 61% for COPER).

#### 3.3.4 Estimated receiver operating characteristic curve (basic symptoms only)

**Fig. 2:**
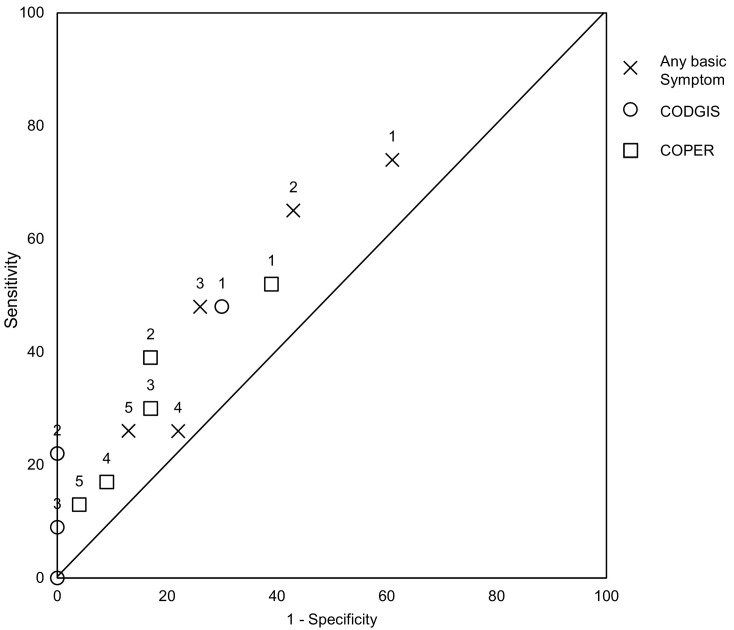
A descriptive ROC curve exploring the predictive value of different basic symptom criteria (data labels indicate threshold number of basic symptoms used to estimate sensitivity and specificity figures).

Fig. 2 illustrates the relative sensitivity and specificity of basic symptoms based on available data from the current study. Sensitivity and specificity were estimated, as described above, for any basic symptom, COPER basic symptoms and COGDIS basic symptoms at thresholds of 1, 2, 3, 4 and 5 basic symptoms present. Predicting relapse using any basic symptom was more sensitive but less specific than using a sub-set such as COPER or COGDIS. Conversely, using a higher threshold (e.g. 2 basic symptoms rather than 1) yielded a more specific but less sensitive assessment of imminent relapse. Fig. 2 gives an approximation of the area within which the ROC (Receiver Operating Characteristic) curves may fall for the three groups of basic symptoms. Although there are no points close to the top left corner of the graph (which would indicate extremely good prediction of relapse), the points do not straddle the line either suggesting that these groups of basic symptoms do have some predictive value. Visual inspection gives an overall impression that using a threshold of ≥ 2 or ≥ 3 basic symptoms, or alternatively ≥ 2 COPER basic symptoms, may give a better balance of sensitivity and specificity than using a threshold of ≥ 1 basic symptoms.

#### 3.3.5 How did people describe basic symptoms?

The eighteen most frequently self-reported basic symptoms are shown in Table 2, with example quotations. A further 15 basic symptoms, not listed in the table, were reported by only one participant each. Thus 73% (33/45) of basic symptoms listed in the SPI-A were specifically identified as beginning or increasing during the period before relapse, rather than at other times. The quotations from participants (Table 2) provide rich, authentic descriptions of basic symptoms, which will be used to design items for a self-report measure.

**Table 2:** Top pre-relapse basic symptoms reported by Sample A participants (n = 23) during the in-depth interview or verbal checklist.

Item (Schizophrenia Proneness Index item number)	Frequency	Example quotation
Increased indecisiveness about insignificant choices (C1)	5	“I don't know what I want half the time. Even going to the shop I can't even choose what sandwich I want.”
Poor at multitasking (B1)^a^	5	“When I'm doing one thing, kind of like my mind gets taken over by that thing and I've got no more room left for other things.”
Thought interference (C2)^a^, ^b^	4	“I was up all night and… I just started having random like weird thoughts.”
Disturbance of receptive speech (C4)^a^, ^b^	3	“It wasn't something that was their issue, it was my cognitive ability to process it really rather than it being they weren't saying things the way that they should have been.”
Increased stress reactivity (A1)	3	[Interviewer: what kind of things would stress you out?] “Talking to anyone, family or friend… couldn't speak to anyone.”
Hypersensitivity to sounds (F4)	3	“I was really noticing birds, and it felt like it was going straight into my head, you know the noise”.
Straight things crooked or double vision (O4.6)^b^	3	“Erm the clock sometimes, it seems a bit bent”; “I mean like it, it's straight but I just sometimes I think it's bent.”; “I thought my eyes must be playing up [laughs]”
Thought perseveration (O1)^b^	3	“Like an annoying thought that you you know like you're trying to focus on something and you really can't focus on it cos you're got this like silly thought in your head.”
Overly distracted by stimuli (B2)	2	“I'll be writing but then I'd get drawn away from it to other things whereas usually…I have a big capacity for concentration solely focusing on one thing and getting it done”
Disturbances of olfactory, gustatory or tactile perception (O6)	2	“Sometimes when I taste food of what other people make then I just, it feels very like, like it's not good, like it's expired or something…it tastes really, like nasty to me the food.”
Micropsia or macropsia (F3)^b^	2	“I remember saying that something looked too big and everyone was saying ‘there's nothing wrong with it, it looks fine’ and I was going ‘it's too big, it's too big, it's too big, it's too big!’.”
Near or tele-vision (O4.1)^b^	2	“I get this feeling everything's distant. And other things are near at hand. This table…it could be nearer or further away; nearer to hand or further away.”
Shapes appear different or distorted (O4.2)^b^	2	“Buildings and people look out of shape.”
Decreased ability to distinguish between ideas and perception or fantasy and true memories (O2)^b^	2	“In things that were almost insignificant really like for example, I'd think oh I made that, I had that meal… then you go into the fridge and it was still there and you hadn't had it at all.”
Derealisation (O8)^b^	2	“The world seems strange to me”; “I'm disconnected from the world.”
Slowed down thinking (B5)	2	“A slow thought would always lead to a fast one.”
Thought blockages (C3)^a^, ^b^	2	“A lot of times my thoughts do get blank and it's taken me a long time remembering.”
Thought pressure (D3)	2	“My thoughts were actually pretty random… my mind was all over the place.”

a Item from Cognitive Disturbances (COGDIS) basic symptoms list.

b Item from Cognitive-Perceptive (COPER) basic symptoms list.

### 3.4 What is the best way of identifying basic symptoms: in-depth interview or verbal checklist?

Twice as many participants reported ≥ 1 basic symptom during the verbal checklist (69.6%) than during the in-depth interview (34.8%), a statistically significant difference (χ^2^ = 6.40, p = 0.022). The number of basic symptoms reported in the verbal checklist (Median = 2, IQR = 0,3) was also significantly higher (z = 2.87, p = 0.004) than in the in-depth interview (Median = 0, IQR = 0,1).

### 3.5 Which pre-relapse experiences are reported in casenotes?

Table 3 shows the percentages of participants for whom each type of pre-relapse experience (basic symptoms, early sign, ‘other’) was reported via self-report (in-depth interviews, verbal checklists) or in casenotes, and the median number of experiences reported in each case. As with the self-reported data, examination of individual items indicated that although a large range of pre-relapse experiences was reported in casenotes, half of these (34/68, 50%) were reported in ≤ 2 participants' casenotes.

**Table 3:** Self-reported (Sample A) and casenote-reported (Samples A and B) pre-relapse experiences (early signs, basic symptoms, ‘other’ pre-relapse experiences).

	Sample A self-report (n = 21)^a^		Sample A casenotes (n = 21)^a^		Sample B casenotes (n = 187)		Comparison of Sample A self-report and casenotes		Comparison of Sample A and Sample B casenotes	
	n	(%)	n	(%)	n	(%)	χ^2^	p	χ^2^	p
≥ 1 reported										
Early signs	21	(100.0)	14	(66.7)	135	(72.2)	7.00	0.016	0.28	0.594
Basic-symptoms	16	(76.2)	1	(4.8)	10	(5.3)	15.00	0.001	0.01	1.000
Other	21	(100.0)	13	(61.9)	106	(56.7)	8.00	0.008	0.21	0.647

	Mdn	IQR	Mdn	IQR	Mdn	IQR	z	p	U	p
Number reported										
Early signs	5	(3,6)	2	(0,4)	1	(0,3)	1.562	0.118	1.31	0.189
Basic symptoms	2	(1,5)	0	(0,0)	0	(0,0)	3.545	0.001	0.06	0.949
Other	4	(3,5)	1	(0,2)	1	(0,2)	3.46	0.001	1.27	0.203

a Statistical comparisons of self-reported and casenote data were only performed for 21 participants as two participants from Sample A did not give consent for their casenotes to be examined. This is why the sample size for Sample A is listed here as 21 rather than 23.

Interviewees were significantly more likely to self-report ≥ 1 early sign, basic symptom or ‘other’ experience, respectively, than was reported in their casenotes (Table 3). The largest difference was for basic symptoms, with sixteen times more participants' self-reporting basic symptoms (76.2% participants) than had them reported in casenotes (4.8%). The number of self-reported basic symptoms and ‘other’ experiences was significantly higher than in casenotes, whereas the reported number of early signs did not differ between self-report and casenotes (Table 3).

Table 3 also compares the two study samples in terms of casenote-reported pre-relapse experiences. The samples did not differ in terms of the proportion reporting each type of pre-relapse experience (basic symptoms, early signs, ‘other’) or the number of these experiences reported. The two samples also did not differ in terms of gender, ethnicity or living situation (see Table 1), but the non-interview sample (B: n = 187) was significantly older and more likely to have high levels of family contact than the interview sample (A: n = 23). Thus, the two samples were largely comparable, with significant differences apparent on only 2 of the 12 assessed variables.

## 4 Discussion

This study used 23 in-depth interview transcripts, 23 verbal checklist transcripts and 208 casenote extracts to: i) examine which basic symptoms occurred prior to relapse; ii) how these basic symptoms were described in order to compare spontaneous and prompted self-reported symptoms; and iii) compare self-reported experiences to those assessed in clinical practice. Three-quarters of participants retrospectively reported ≥ 1 basic symptom that began or increased prior to a recent relapse. All participants reported ≥ 1 conventional early sign and ‘other’ pre-relapse experience. Although participants reported significantly more early signs than they did basic symptoms, a third of participants who reported basic symptoms reported as many as they did conventional early signs. Participants gave rich descriptions of basic symptoms, but only when prompted.

Basic symptoms have been shown to predict FEP in continental European samples (Fusar-Poli et al., 2012, Schultze-Lutter et al., 2007). Only two previous studies (Bechdolf et al., 2002, Gaebel and Riesbeck, 2014) have investigated whether basic symptoms occurred prior to relapse in those with established psychosis. A small, retrospective study (Bechdolf et al., 2002) found basic symptoms reported prior to both depressive and psychotic episodes, with differences in the content of basic symptoms distinguishing a depressive from a psychotic episode. Unlike the current study, only those with ICD-10 paranoid schizophrenia who had no residual symptoms were included in the psychosis sample, which limits the generalizability of the findings. More recently, a larger study (Gaebel and Riesbeck, 2014) appears to have prospectively assessed basic symptoms in those with a previous episode of psychosis. Although the authors do not explicitly state that they examined basic symptoms, they list ten items whose brief descriptions resemble the COPER sub-scale of the SPI-A. Sensitivity (mean 12%) and specificity (mean 97%) values for these 10 individual items provide some evidence that basic symptoms occur prior to relapse. However, since no combined predictive value for the COPER-like items is given, one cannot draw strong conclusions about the value of basic symptoms as predictors of relapse.

The current study shows that one can identify pre-relapse basic symptoms in a UK sample of patients with chronic schizophrenia. Although the current retrospective study cannot determine definitively how well basic symptoms predict relapse, it does indicate that it is a question worth further investigation. A prospective study comparing the sensitivity and specificity of basic symptoms and conventional early signs is warranted. Data from the current study will inform the development of a definitive prospective study in three specific ways: i) it will help to determine the threshold number (and type) of basic symptoms required to count someone as a ‘case’ (i.e. predicted to have a relapse); ii) it will inform the design of a self-report measure for prospectively monitoring basic symptoms; iii) it lends weight to the idea that monitoring an individualised ‘relapse signature’ is the most efficient way of spotting early indicators of relapse (given the wide range and idiosyncratic nature of reported pre-relapse experiences).

Estimated retrospective sensitivity of reporting ≥ 1 basic symptom (74%) in the current study was higher than the median prospective sensitivity (61%) of early signs measures in a recent review (Eisner et al., 2013), but estimated specificity was lower (current study, 39%; review median, 81%). This suggests that using ≥ 1 basic symptom is not the optimum threshold for caseness. Although there was insufficient data to perform a formal ROC analysis with probability testing, we plotted a descriptive ROC curve to explore whether setting a higher threshold for caseness (e.g. ≥ 3 basic symptoms) or defining a smaller, more predictive set of basic symptoms (e.g. COGDIS or COPER) would provide a better balance of sensitivity and specificity. Visual inspection of the ROC curve suggested that a threshold of ≥ 2 or ≥ 3 basic symptoms, or alternatively ≥ 2 COPER basic symptoms, may give a better balance of sensitivity and specificity.

To monitor basic symptoms as part of a personalised relapse signature, clinicians need to identify which of these an individual experienced prior to previous relapses. The best way of identifying pre-relapse basic symptoms in the current study was a verbal checklist asking specifically about these experiences: twice as many participants identified basic symptoms during verbal checklists than during in-depth interviews. Firstly, this may be because the in-depth interview required recall memory whereas the verbal checklist only used recognition memory, the latter being easier (Speer and Flavell, 1979) and both being impaired in schizophrenia (Libby et al., 2013). Secondly, unlike psychotic symptoms, patients are not commonly asked about basic symptoms in a UK mental health service context. Aside from a small number of Early Intervention Services assessing basic symptoms as prodromal symptoms of FEP (e.g. Lancashire Early Assessment and Detection Clinic; Johnson, 2013), we know of very few clinical services in the UK who assess them. Participants in the current study (all receiving care from UK mental health services) may not have spontaneously divulged these experiences because they did not expect the interviewer to be interested in them. Thirdly, patients may not recognise the experiences as ‘symptoms’ per se and only begin to think their experience is unusual when asked about them. Fourthly, some basic symptoms may not be spontaneously divulged due to embarrassment, whereas being asked as part of a checklist, framed as a list of experiences that people sometimes report before relapse, may normalise the experience.

The casenote data gives an insight into which pre-relapse experiences are assessed in current practice. Only 5% of casenotes contained ≥ 1 basic symptom, with participants sixteen times more likely to self-report a basic symptom than the casenotes. This was as predicted: basic symptoms are not currently enquired about in British psychiatry, especially not as early indicators of relapse, and clinicians are not generally trained to assess them. Furthermore, basic symptoms are subtle and subjective, without outward signs that they are occurring, and patients tend not to disclose them until prompted.

This study used directed content analysis to quantify the number of basic symptoms, early signs and ‘other’ experiences reported during in-depth interviews, verbal checklists and in casenotes. Although the coding method was systematic, it was necessary for the coder to use judgement at times. Nevertheless inter-rater reliability, assessed in 10% of cases, was generally high. For all statistical analyses, findings were considered significant at p = 0.05. Using a different threshold for significance may have resulted in different conclusions. There are limitations specific to the self-reported data. Firstly, the sample for this data was relatively small and purposively rather than randomly selected. We used non-parametric statistics to account for the resultant non-normality of the data and compared the purposive sample to a much larger, randomly selected sample. Since the two samples were largely comparable, it is likely that the purposive sample provides a fairly good representation of the experiences of this patient group. Secondly, data was gathered retrospectively, which may have introduced bias; to minimize this, only patients who had relapsed in the past six months were interviewed. Thirdly, inherent in all studies where any two measures are used serially, the first measure could prime the second. We aimed to minimize this effect by doing the in-depth interview, with its open questions, first and the verbal checklist of basic symptoms second. Fourthly, there were limitations of the descriptive ROC curve (Fig. 2) and the specificity data upon which it is based. There was insufficient data to perform a formal ROC analysis with probability testing; the estimates of specificity are likely to be biased since they were based on incidental reports during assessments aiming to elicit pre-relapse experiences. Where high specificity is shown, this may be due to lack of data rather than a genuinely highly specific assessment.

In summary, most interviewees self-reported pre-relapse basic symptoms but very few casenotes reported them. Basic symptoms may be potent predictors of relapse that clinicians miss. The best way of identifying pre-relapse basic symptoms was a verbal checklist asking specifically about these experiences. Use of a basic symptoms checklist in clinical practice, in conjunction with an existing checklist of conventional early signs, may yield a richer relapse signature. A prospective study examining whether adding basic symptoms to conventional early signs of relapse enhances predictive value is warranted. A self-report measure of basic symptoms could facilitate such a prospective investigation and aid monitoring of basic symptoms in routine clinical practice.

## Appendix A Supplementary data

Additional rationale for study research questions and additional methods. Image 1
