# Supplementary material for: Comparing early signs and basic symptoms as methods for predicting psychotic relapse in clinical practice
Source: Schizophr Res. 2018 Feb;192:124–30. doi: 10.1016/j.schres.2017.04.050 (PMC5821684; doi:10.1016/j.schres.2017.04.050)
Supplement: Supplementary file 1 — Additional rationale for study research questions and additional methods. [file mmc1.docx]

**Supplementary Material**

**Section A: Additional rationale for study research questions**

*Research question 1: Which pre-relapse experiences (early signs, basic symptoms, ‘other’) do participants report and how do they describe them?*

We aimed to investigate whether basic symptoms could be used to predict relapse in routine clinical practice and to compare them to conventional early signs in anticipation of developing and prospectively testing a basic symptoms measure. A new measure of basic symptoms is necessary for future prospective investigations of basic symptoms as possible early indicators of relapse since existing interview-based measures such as the SPI-A ([Schultze-Lutter et al., 2007](#_ENREF_30)) are time consuming and not relapse-specific. To inform the measure’s design we recorded how basic symptoms were described by patients.

*Research question 2: What is the best way of identifying basic symptoms: in-depth interview or verbal checklist?*

The best method of identifying pre-relapse basic symptoms in clinical practice has not yet been addressed in the literature. Conventional early signs are commonly identified by answering open questions in a semi-structured interview ([Tait et al., 2002](#_ENREF_34)) and/or completing a checklist indicating whether or not a number of pre-determined early signs have been experienced ([Marder et al., 1994](#_ENREF_26)). This study compares the number of basic symptoms identified using these two approaches. Their relative merits are currently unclear since a checklist may be quicker than an interview but less accurate.

*Research question 3: Which pre-relapse experiences (early signs, basic symptoms, ‘other’) are reported in casenotes?*

In clinical practice, early signs monitoring tends to be personalised, with the patient and/or clinician defining a ‘relapse signature’ comprising a small number of early signs that occurred prior to previous psychotic episodes ([Birchwood et al., 2000](#_ENREF_8)). The relapse signature could include basic symptoms, but our clinical experience suggests that UK clinicians would not include these. Therefore we compared self-reported pre-relapse experiences with those reported in participants’ casenotes to give an indication of the extent and accuracy of current clinical practice in identifying conventional early signs, basic symptoms and other pre-relapse experiences.

**Section B: Additional methods**

***Directed content analysis***

*Overview*

To quantify pre-relapse experiences, the research team conducted a directed content analysis ([Hsieh and Shannon, 2005](#_ENREF_20)), facilitated by Nvivo-9. This method aims to validate or conceptually extend a theoretical framework and uses existing theory or research within the analysis process, e.g. to determine the initial coding scheme ([Hsieh and Shannon, 2005](#_ENREF_20)).

*Stage-of-relapse coding*

All transcripts were initially coded by the first author according to the stage of the relapse process being described (pre-relapse, during relapse, unrelated to relapse). The first and second author then reached a consensus opinion by discussing detailed chronological summaries of participants’ deteriorations.

*Pre-relapse experience coding*

Pre-relapse experiences reported in in-depth interview transcripts, verbal checklist transcripts and casenote extracts were coded by the first author in discussion with members of the research team (CB, RD). The initial coding scheme consisted of conventional early signs (34 ESS items) and basic symptoms (56 SPI-A items). For experiences considered to be early indicators of relapse that were not codable using existing items, new codes were defined and assigned to an ‘other’ category. These could be feelings, thoughts, behaviours or internal experiences that changed during the few months prior to relapse. Long-term experiences that had not changed prior to relapse were excluded from the ‘other’ category, as were external circumstances (e.g. life events, external stressors), covered in a companion paper ([Eisner et al., 2014](#_ENREF_11)). Once all data was coded, overlapping codes were combined, resulting in 119 codes (45 basic symptoms, 23 early signs, 51 ‘other’). Overlap between early signs and basic symptoms codes was resolved by retaining the early signs code and combining the basic symptoms code with it.

*Inter-rater reliability*

**Casenote data extraction:** reliability was assessed in terms of percentage agreement with consensus extraction. **Stage-of-relapse coding**: the third author independently coded the detailed chronological summaries of deterioration. This was compared with the first and second authors’ consensus coding of the same summaries using weighted kappa. **Pre-relapse experience coding**: the fourth author coded a randomly selected subset (12%) of the data, stratified by data type. This was compared with the first author’s coding, using intra-class correlations (ICC; number of items endorsed) and Cohen’s kappa (≥1 item endorsed).

***Statistical analysis***

*Q1: Which pre-relapse experiences do participants report?*

The numbers of self-reported (in-depth interview, verbal checklist) basic symptoms and early signs were compared within-subjects using Wilcoxon signed-rank test in the interview sample (n=23). An exploratory analysis examined whether those self-reporting basic symptoms differed from others demographically or clinically (Mann-Whitney, Chi-squared test).

*Q2: What is the best way of identifying basic symptoms?*

Two methods of eliciting basic symptoms (in-depth interviews and verbal checklists) were compared within-subjects in the interview sample (n=23), using McNemar’s test (dichotomised to whether ≥1 basic symptom reported) or Wilcoxon signed-rank test (number of basic symptoms reported).

*Q3: Which pre-relapse experiences are reported in casenotes?*

Self-reported (in-depth interview, verbal checklist) and casenote data were compared in terms of reported early signs, basic symptoms and ‘other’ experiences (McNemar’s test, Wilcoxon signed-rank test). To determine the extent to which Samples A & B were similar, their demographic and clinical characteristics and the number of casenote basic symptoms, early signs and ‘other’ pre-relapse experiences were compared (Chi-squared or Mann-Whitney test).
